# Low-Molecular-Weight Heparin Versus Warfarin in Adult Cancer Patients as a Precision Medicine for Thrombosis: A Systematic Review and Meta-Analysis

**DOI:** 10.7759/cureus.41268

**Published:** 2023-07-01

**Authors:** Hany A Zaki, Baha Hamdi Alkahlout, Kaleem Basharat, Wael Abdelrehem Elnabawy Elsayed, Mohammed Gafar Abdelrahim, Nood Dhafi R Al-Marri, Maarij Masood, Eman Shaban

**Affiliations:** 1 Emergency Medicine, Hamad Medical Corporation, Doha, QAT; 2 Cardiology, Al Jufairi Diagnosis and Treatment, Doha, QAT

**Keywords:** malignancy, recurrent deep vein thrombosis, recurrent venous thromboembolism, enoxaparin, dalteparin, low-molecular weight heparin, vitamin k antagonists, warfarin

## Abstract

Venous thromboembolism (VTE) is a condition often seen in patients diagnosed with cancer and is recognized as a predictor of poor outcomes in these patients. The probability of VTE recurring is generally higher in people with cancer than in those without; hence, addressing this issue is essential when making healthcare decisions. Therefore, our systematic review was primarily designed to compare low-weight- molecular heparin (LMWH) to warfarin in reducing recurrent VTE among cancer patients. However, other outcomes were also evaluated, such as mortality and bleeding events observed more in cancer patients.

The selection of relevant articles was carried out using a database search and a manual search, which involved reviewing reference lists of articles eligible for inclusion in the current review. The methodological quality of each included study was then assessed using Cochrane’s risk of bias tool in the Review Manager software (RevMan 5.4.1). Additionally, pooled results were examined using the Review Manager software and presented as forest plots.

Our search of electronic databases elicited a total of 2163 articles, of which only six were deemed eligible for inclusion and analysis. Data pooled from the six studies demonstrated the effectiveness of LMWH in minimizing the reoccurrence of VTE over warfarin [risk ratio (RR): 0.67; 95% CI: 0.47 - 0.95; p = 0.03]. However, LMWH had a similar effect statistically as warfarin on the major bleeding events (RR: 1.05; 95% CI: 0.62 - 1.77; p = 0.85), minor bleeding events (RR: 0.80; 95% CI: 0.54 - 1.20; p = 0.28), and all-cause mortality (RR: 1.00; 95% CI: 0.88 - 1.13; p = 0.99).

While LMWH demonstrated its effectiveness in minimizing the incidence of VTE recurrence over warfarin in cancer patients, it had no statistical difference in terms of mortality or bleeding events when compared to warfarin. Based on our findings, we recommend that LMWH continues to be used as a first-line treatment regimen to mitigate recurrent VTE in cancer patients.

## Introduction and background

Venous thromboembolism (VTE) is a condition that often manifests in patients diagnosed with cancer and is recognized as a predictor of poor outcomes in these patients [1-3]. Research has found that the probability of VTE recurring in cancer patients is three times that of non-cancer patients [3]. Moreover, research reveals that the mortality risk in cancer patients with VTE is three to eight times greater than those without cancer [4]. Hence, proper management and prevention therapies for recurrent VTE are crucial in the care of cancer patients. The risk of VTE in this study population increases based on various etiologies, including surgical operations, chemotherapy, hormonal agents, immobilization, insertion of the central venous catheter, and the prothrombotic and fibrinolytic nature of the tumor cells [3].

Healthcare practitioners have recently come to prefer low-molecular-weight heparin (LMWH) as the first-line drug regimen for mitigating recurrent VTE in individuals with cancer. The American College of Chest Physicians has proposed that cancer patients should receive LMWH for mitigating VTE over vitamin K antagonists (VKAs) and other direct oral anticoagulants (DOACs) [5]. While the focus has been on LMWH, the substantial injection costs, patients’ tendency to choose a particular drug, and renal issues have pushed healthcare practitioners to explore the safety and effectiveness of oral anticoagulants for treating and preventing recurrent VTE in cancer patients. Warfarin has been considered an alternative to LMWH, and earlier research papers have suggested that it may be as safe and effective as DOACs in combating VTE among cancer patients [6-8]. Nevertheless, a meta-analysis that directly compares warfarin to LMWH in reducing recurrent VTE in malignancy patients has yet to be undertaken. In light of this, we conducted the present research to compare the prevalence of cancer-related recurrent VTE between patients receiving warfarin and those on LMWH. Furthermore, our study will assess the incidence of bleeding episodes and all-cause mortality in these patients.

## Review

Materials and methods

Eligibility Criteria

One of the reviewers with prior expertise in examining eligibility criteria devised a set of conditions for including and excluding research articles. Our inclusion criteria were as follows: randomized trials and observational studies published in English (this criterion was a vital part of our study because it helped us to avoid direct translations of scientific terms that would otherwise influence our scientific purpose); studies that included cancer or malignancy patients aged 18 years and above; studies that directly compared an LMWH with warfarin; studies in which warfarin was administered after LMWHs were used to achieve a stable international normalized ratio (INR) of 2-3; studies with sufficient sample sizes, i.e., more than 30 patients this criterion was vital in ensuring that our meta-analyses had sufficient statistical power); and studies that reported the incidence of recurrent VTE between the study regimens or bleeding events and mortality.

Conversely, our exclusion criteria were as follows: letters to the editor, guidelines, abstracts without full articles, systematic reviews, cost-effective analyses, case series, case reports, and studies individually comparing either LMWH or warfarin to other drug regimens such as DOACs.

Literature Search

One reviewer searched for literature relevant to our research topic by adhering to the Preferred Reporting Items for Systematic Reviews and Meta-Analyses (PRISMA) guidelines. The search involved using specific keywords on five electronic databases (PubMed, ScienceDirect, Medline, Google Scholar, and Scopus) and reviewing the reference lists of studies from these databases. The keywords employed in the electronic databases were as follows: (“Warfarin” OR “coumarin derivatives” OR “Vitamin K antagonist” OR “VKA”) AND (“low molecular weight heparin” OR “LMWH” OR “tinzaparin” OR “Dalteparin” OR “Ardeparin” OR “Enoxaparin” OR “Nadroparin” OR “Reviparin”) AND (“recurrent venous thromboembolism” OR “recurrent VTE” OR “recurrent deep vein thrombosis” OR “recurrent pulmonary embolism”) AND (“malignancy” OR “Cancer”) AND (“Adults”). During the search, we made it a point to avoid all close or exact duplicates and gray literature since they would have undermined the scientific research purpose of our study. In addition, no limitation was placed on the publication year since we wanted to expand our scientific research.

Data Extraction and Definitions

The data extraction process was carried out by two reviewers, who then summarized the data in a tabular form. The data retrieved from each article comprised author ID (surname of the first author and year in which the study was first published), the country in which the study was conducted, design of the study, participants’ characteristics, warfarin and LMWH dosages, follow-up period, and the main outcomes. The primary endpoint of our review was the prevalence of recurrent VTE, while secondary endpoints were all-cause mortality and bleeding episodes. All the inconsistencies in retrieved data were addressed by interactive dialogue between the two reviewers or by contacting a third author who functioned as an arbitrator.

Bleeding events were categorized into major and minor events. Based on the descriptions by the International Society on Thrombosis and Haemostasis (ISTH), bleeding was referred to as “major” if it was clinically preceded by at least a 2 g/dL drop in the levels of hemoglobin or at least two units of packed red blood cells transfusion, and mainly occurred at critical sites or resulted in fatality or hospitalization [9]. Minor bleeding was defined as any bleeding episode that did not match the conditions for major bleeding.

Quality Appraisal

**Figure 1:**
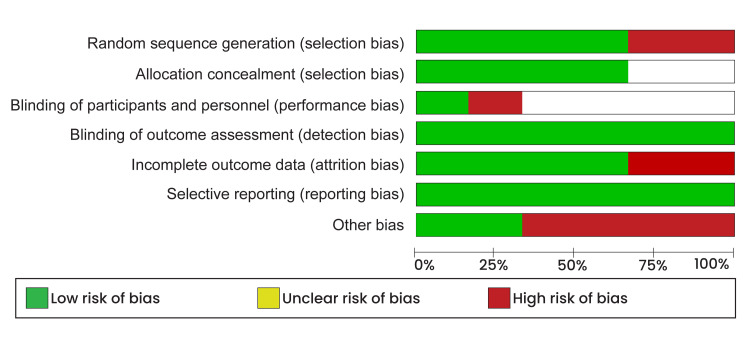
Risk of bias graph

**Figure 2:**
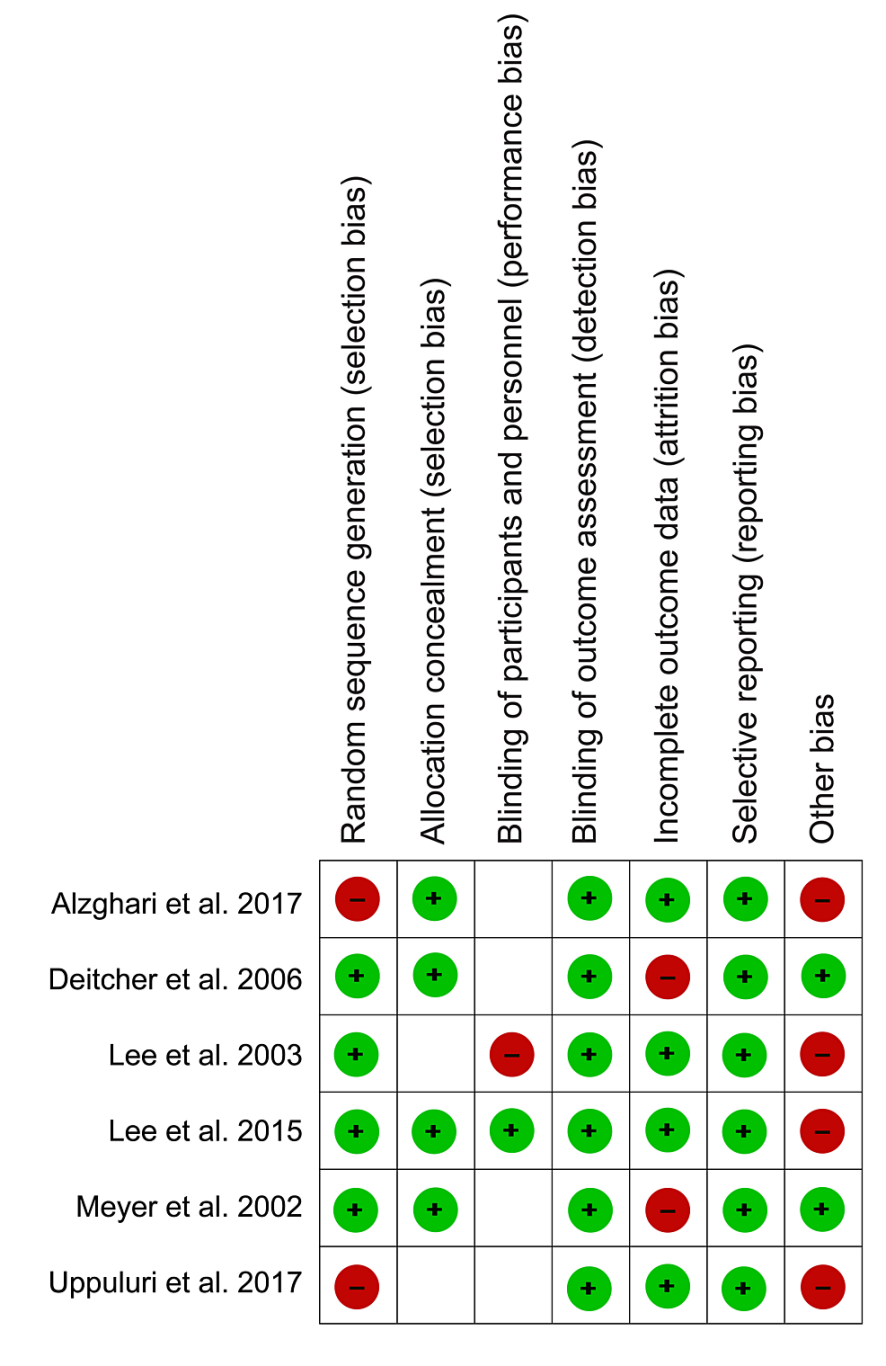
Risk of bias summary* *[10-15]

Our study was designed as a comparative interventional review; therefore, methodological quality was appraised using Cochrane’s risk of bias tool from the Review Manager Software (RevMan 5.4.1). This quality appraisal method utilized four assessment criteria (selection, attrition, performance, and reporting bias) and color codes to identify the risk of bias. The green color was used for a low risk of bias, which meant that the assessment criteria were fully answered, while red was used for a high risk of bias, which meant that the assessment criteria were not addressed. Conversely, the unclear risk of bias was not assigned a color code and was used when the reviewers could not judge a specific criterion due to a lack of details. The risk of bias assessment results are presented as a risk of bias graph (Figure 1), and the risk of bias summary is presented in Figure 2).

Data Synthesis

The overall effect sizes and differences between the rates of recurrent VTE, all-cause mortality, and bleeding events were calculated using the Review Manager software (RevMan 5.4.1). The data for all outcomes in this review were dichotomous; therefore, we employed the risk ratio (RR), a random effect model, and a 95% confidence interval for all calculations. Moreover, we calculated the heterogeneity between the studies using I^2^ statistics, in which values between 0 - 49, 50 - 69, and 70 - 100 were regarded as low, moderate, and high, respectively. The final results were then presented as forest plots of significance and were estimated using p-value, with the level of significance set at p<0.05.

Results

Selection Criteria

**Figure 3:**
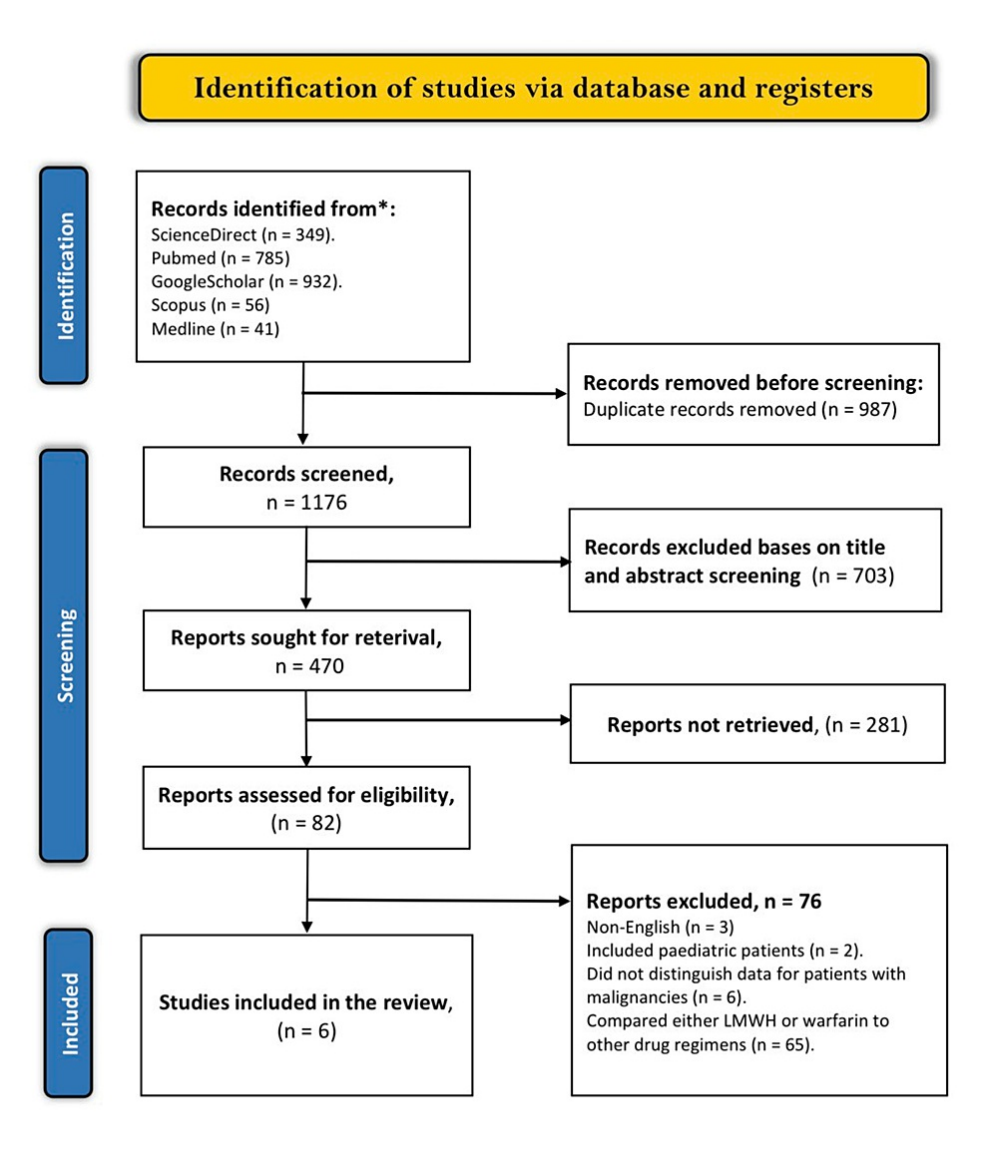
PRISMA flow diagram depicting the literature search results PRISMA: Preferred Reporting Items for Systematic Reviews and Meta-Analyses

The literature search on the electronic databases yielded 2163 articles based on the specified keywords. We carried out a duplicate analysis of these articles, and 987 articles identified as either close or exact duplicates were excluded from the review. The remaining articles were screened based on their titles and abstracts, and 703 articles were excluded as they did not meet the screening criteria. Out of the remaining 470 studies, 388 were not selected because they were either ongoing trials, abstracts without full evidence, letters to the editor, Guidelines, case reports, cost-effective analyses, or systematic reviews. At the end of the search process, only six articles were found to have met the criteria for inclusion. The other 76 articles were excluded for the following reasons: three were non-English articles, two included pediatric patients, 65 individually compared either LMWH or warfarin to different drug regimens, and six included patients with other etiologies of recurrent VTE but did not distinguish data for patients with malignancies. A summary of the full selection criteria is shown in the PRISMA flow diagram below (Figure 3).

The characteristics of the included studies are summarized in Table 1.

**Table 1:** Study characteristics RCT: randomized controlled trial; VTE: venous thromboembolism; LMWH: low-molecular-weight heparin; INR: international normalized ratio; NR: not reported

Author ID	Study design	Location	Participant characteristics	LMWH dosage	Warfarin dosage	Follow-up (months)	Main outcomes
Meyer et al., 2002 [10]	Open-label multicenter RCT	France	146 patients (65 males and 81 females)	A fixed dose of 1.5 mg/kg per body weight of enoxaparin for 3 months	1.5 mg/kg of enoxaparin every 24 hours for at least 4 days until an INR of at least 2 was achieved, then 6 to 10 mg of oral warfarin was given for 3 months and adjusted to reach INR of 2 to 3	3 and 6	At 3 months, 17 patients who had received warfarin died compared to 8 who received enoxaparin. During the 3-month follow-up, VTE reoccurred in 15 warfarin-treated patients compared to 7 enoxaparin-treated patients. Major bleeding was recorded in 12 patients who had received warfarin compared to 5 patients who received enoxaparin. At 6 months, minor bleeding was recorded in 9 patients who received warfarin compared to 5 who received enoxaparin
Lee et al., 2015 [11]	Open-label multinational RCT	Asia, Africa, Europe, and North, Central, and South America	900 patients (535 females and 365 males)	Innohep® (tinzaparin sodium) 20,000 anti-Xa IU/ml dispensed in 0.5, 0.7- and 0.9-ml syringes	Warfarin tablets of 1, 3, and 5 mg for 6 months to maintain the therapeutic INR and initial treatment with Innohep® for 5 – 10 days	6	VTE reoccurrence was recorded in 31 tinzaparin-treated patients compared to 45 warfarin-treated patients. All-cause mortality was recorded in 150 patients who received tinzaparin compared to 138 who received warfarin. Major hemorrhage befell 12 patients in whom tinzaparin was administered patients and 11 patients in whom warfarin was administered, while minor bleeding was recorded in 49 and 69 patients, respectively
Deitcher et al., 2006 [12]	Open-label parallel RCT	United States	101 patients (mean age: 63.7 ± 12.0 years)	Subcutaneous 1.0 mg/kg of enoxaparin twice per day for 5 days, followed by 1.0 mg/kg of enoxaparin once daily for 175 days. OR Subcutaneous 1.0 mg/kg of enoxaparin twice per day for 5 days, followed by 1.5 mg/kg of enoxaparin once daily for 175 days	Subcutaneous 1.0 mg/kg of enoxaparin twice per day for at least 5 days until INR of 2 to 3 was achieved, and oral warfarin started at day 2 of enoxaparin administration for a total of 180 days continuously	6	Recurrent VTE was recorded in 1 patient receiving 1.0 mg/kg of enoxaparin, 1 patient receiving 1.5 mg/kg of enoxaparin, and 2 patients receiving warfarin. Major bleeding was recorded in 19, 20, and 17 patients receiving 1.0 mg/kg of enoxaparin, 1.5 mg/kg of enoxaparin, and warfarin, while minor bleeding was recorded in 2, 4, and 1 patients, respectively. All-cause mortality was recorded in 7, 15, and 11 patients receiving 1.0 mg/kg of enoxaparin, 1.5 mg/kg of enoxaparin, and warfarin respectively
Alzghari et al., 2017 [13]	Retrospective study	United States	127 patients (69 females and 58 males)	Enoxaparin for 3 months	Oral warfarin for at least 75% of the total time on anticoagulants	6	At 6 months, recurrent VTE was recorded in 5 patients receiving enoxaparin and 7 receiving warfarin. At 6 months, cases of major bleeding were recorded in 1 patient receiving enoxaparin and 4 receiving warfarin; 13 of 23 receiving enoxaparin died, while 13 of 56 patients receiving warfarin died
Uppuluri et al., 2017 [14]	Retrospective cohort study	United States	131 patients (82 females and 49 males)	NR	NR	1	Recurrent VTE was recorded in 8 patients receiving LMWH compared to 2 patients receiving warfarin. Minor bleeding was recorded in 5 patients receiving LMWH compared to 1 patient receiving warfarin, while major bleeding was recorded in 5 and 1 patient, respectively; 23 of 86 patients receiving LMWH died compared to 1 of 34 patients receiving warfarin
Lee et al., 2003 [15]	Open-label multicenter RCT	Canada, Australia, New Zealand, the United States, Italy, Spain, the United Kingdom, and the Netherlands	676 patients (348 females and 328 males)	200 UI/kg of subcutaneous dalteparin from multidose vials once daily for 1 month, and for the remaining 5 months, patients were treated with 75 – 83% of the full dose (approximately 150 UI/kg)	200 IU/kg per body weight of subcutaneous dalteparin once daily for at least 5 days until an INR of 2.5 was achieved, and within 24 hours of dalteparin administration, oral warfarin was administered until 6 months	6	27 patients receiving dalteparin had recurrent VTE compared to 53 patients receiving oral anticoagulants. Major bleeding was recorded in 19 of 338 patients who received dalteparin and 12 of 335 patients who received oral anticoagulants; 130 patients receiving dalteparin died compared to 136 patients who received oral anticoagulants

Recurrent VTE

**Figure 4:**
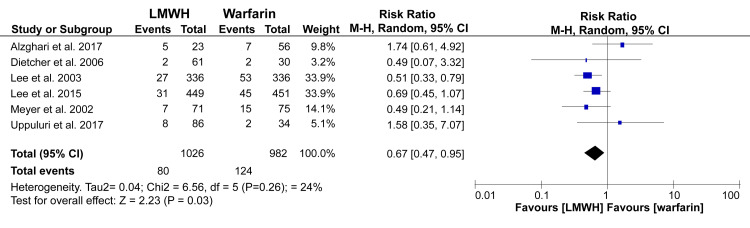
Forest plot - prevalence and 95% confidence interval comparing episodes of recurrent VTE between LMWH and warfarin* *[10-15] VTE: venous thromboembolism; LMWH: low-molecular-weight heparin

Episodes of recurring VTE were reported in all articles in the current review. Most of these incidences were recorded within three to six months of follow-up. However, for our analysis, we grouped all the outcomes during the final follow-up period for each study. The pooled data, irrespective of the timing of outcome assessment, showed that LMWH was statistically more beneficial in reducing episodes of recurrent VTE than warfarin (RR: 0.67; 95% CI: 0.47 - 0.95; p = 0.03) (Figure 4).

Safety Outcomes

**Figure 5:**
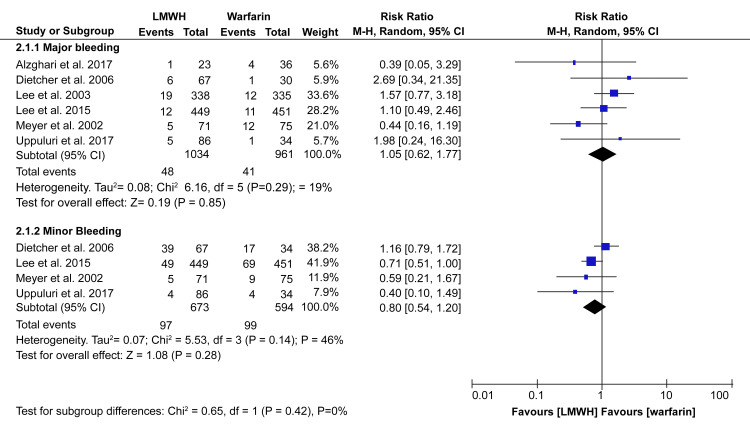
Forest plot - prevalence and 95% confidence interval comparing incidences of bleeding events between LMWH and warfarin* *[10-15] LMWH: low-molecular-weight heparin

The safety of LMWH and warfarin was assessed by analyzing the incidence of bleeding events and all-cause mortality. Pooled data from the studies showed no significant difference between LMWH and warfarin for major bleeding (RR: 1.05; 95% CI: 0.62 - 1.77; p = 0.85) or minor bleeding (RR: 0.80; 95% CI: 0.54 - 1.20; p = 1.20; p = 0.28) (Figure 5).

**Figure 6:**
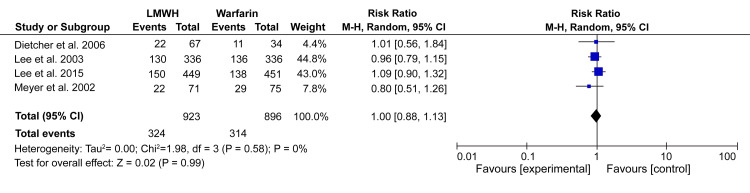
Forest plot - prevalence and 95% confidence interval comparing incidences of all-cause mortality between LMWH and warfarin8 *[10-12,15] LMWH: low-molecular-weight heparin

On the other hand, mortality is a very sensitive issue when assessing the safety of any drug regimen. Therefore, data for mortality was only pooled from the four randomized trials. The pooled analysis showed no significant difference in the risk of death between LMWH and warfarin (RR: 1.00; 95% CI: 0.88 - 1.13; p = 0.99) (Figure 6).

Moreover, our analysis showed no heterogeneity (I^2^ = 0%) between the studies, meaning that the outcomes were highly reliable.

Discussion

Individuals with malignancy are frequently at a greater risk of recurrent thrombotic events and hemorrhage during anticoagulant therapy [16]. Moreover, VTE is a major contributor to death and morbidity [17]. The present research examined the effectiveness of LMWH and warfarin in patients diagnosed with malignancies and discovered that LMWH was considerably more useful in decreasing occurrences of recurrent VTE than warfarin. However, no difference was detected in the overall prevalence of bleeding episodes and all-cause fatalities.

Our findings align with those of three previous systematic reviews that compared LMWH with VKAs (including warfarin) [18-20]. Thein et al. undertook a meta-analysis of six RCTs with 2196 cancer patients and showed that LMWH considerably lowered the probability of VTE recurrence than VKA (RR: 0.572; 95% CI: 0.436 - 0.750; p<0.001). Conversely, the research demonstrated a negligible difference in major bleeding risk (RR: 1.049; p = 0.812) but not minor bleeding (RR: 0.773; p = 0.014), and LMWHs were deemed more beneficial in the reduction of minor bleeding episodes over VKAs. However, this conclusion may have been impacted by the effect of other VKAs, such as acenocoumarin [18]. Similarly, Louzada et al.'s study revealed the significant benefit of LMWHs over VKAs in reducing recurrent VTE events (RR: 0.53; p = 0.0007) but not minor or major bleeding [20]. These findings are further substantiated by a Cochrane systematic review that illustrated that LMWHs are preferable to VKAs in minimizing recurrent VTE episodes but not incidents of hemorrhage [19]. While these studies indicate the superiority of LMWHs over VKAs, including warfarin, evidence in other research implies that warfarin may be equally efficient as LMWHs in the reduction of recurrent VTE events. For example, a recent United States database claims study of 14,086 cancer patients with VTE complications revealed that the adjusted prevalence of recurrent VTE was similar between the 4585 patients who had received warfarin and the 6108 receiving LMWH (HR: 0.91; p = 0.421) [21]. Unfortunately, the data reported in this study cannot be utilized to guide the therapeutic treatment of cancer patients due to multiple limitations. Firstly, this research was designed as a retrospective study, which has an inferior quality of evidence, meaning that only associations can be derived from the study rather than causations. Secondly, inpatient claims were used to define recurrent VTE and this might have impacted their findings. Moreover, the study used duplicate data in their analysis, suggesting that their conclusions might have been biased.

Despite grouping all the LMWHs in one arm, evidence suggests that different LMWHs may have different outcomes in cancer patients compared with warfarin. The CATCH trial examined the efficacy of LMWH (tinzaparin) and warfarin and noticed there was no difference in the prevalence of recurrent VTE in either arm (6.9% vs. 10%, respectively; p = 0.07) [11]. However, the study recorded a lower incidence of recurrent symptomatic deep vein thrombosis (DVT) in the tinzaparin arm than in the warfarin arm (2.7% vs. 5.3%, respectively; p = 0.04). Moreover, the study reported a significantly lower incidence of clinically relevant non-major bleeding events in the tinzaparin group than in warfarin (10.9% vs. 15.3%, respectively; p = 0.004). On the other hand, the CLOT trial used the LMWH dalteparin and found that it significantly reduced the rate of recurrent VTE than warfarin (HR: 0.48; p = 0.002) [15]. We assumed that the outcomes recorded in these trials might have been influenced by the type of regimen used for the secondary prevention of VTE; however, other factors such as associated anticancer treatments, geographic distribution, and tumor sites may have contributed to the discrepancies in the data. Therefore, future randomized trials should compare various LMWHs for the reduction of recurrent VTE to validate our hypothesis fully.

As stated earlier, mortality is a sensitive issue that should be analyzed cautiously. Data from four randomized trials in our study has shown that the risk of all-cause mortality is similar in LMWH and warfarin arms. This finding is consistent with previous meta-analyses comparing LMWHs and VKAs [18-20]; however, data from some studies seem to provide contradictory information. For example, Chiasakul et al. carried out a retrospective cohort study to compare the overall survival rate in cancer-associated thrombosis patients receiving either warfarin or LMWH and found that warfarin was significantly associated with improved overall survival rate than LMWH in the treatment of VTE associated with cancer (HR: 0.86; 95% CI: 0.83 - 0.90; p<0.001) [22]. Similarly, Uppuluri et al. revealed that the mortality rate at six months was higher in the LMWH arm than in the warfarin arm (56.5% vs. 23.2%, respectively). However, it is worth noting that these studies were non-randomized; therefore, randomized trials are required to support their results. Moreover, most of the studies have reported that the most frequent cause of death in this population was cancer progression. For instance, in the CATCH trial, progression in cancer accounted for 69% of overall deaths (23.4% in patients receiving tinzaparin and 20.6% in patients receiving warfarin). Similarly, the randomized trial by Meyer et al. reported high mortality rates due to the progression of cancer: out of 14 patients who died due to the cancer progression, nine had received warfarin compared to the five who received enoxaparin.

Apart from the fact that LMWHs have been demonstrated to be more beneficial in reducing incidences of recurrent VTE, they have other benefits in the long-term therapy of VTE. Firstly, frequent laboratory monitoring is not necessary for LMWH medication because it has a bioavailability that is very predictable preceding subcutaneous administration and a renal clearance that depends on the administered dose [23,24]. Additionally, its anticoagulant action is neither altered by the change in diet nor the usage of concurrent medications, implying that outpatient treatment of thromboprophylaxis with LMWHs is exceptionally feasible [24]. This advantage is deemed more favorable for cancer patients with poor quality of life and minimized hospital visitations in this research group. Furthermore, LMWHs tend to have a more swift action after initiation and clearance that is more predictable compared to warfarin, meaning that they provide more consistent anticoagulation in the course of the treatment and offer more flexibility than warfarin where treatment has to be interrupted for invasive procedures [24,25]. LMWHs are also beneficial in cancer patients because they might be efficient in individuals who develop thrombosis irrespective of therapeutic anticoagulation levels with warfarin, a scenario that is exceedingly observed in cancer patients as opposed to those without cancer [23-25].

Additionally, our analysis showed that LMWHs are delivered subcutaneously instead of orally. Therefore, the challenges of administering an appropriate dosage of anticoagulant orally in vomiting or anorexia patients (frequent complications of cancer and its treatment) are addressed. In addition, studies have shown that the inconveniences associated with the subcutaneous injection of LMWHs may be mitigated by self-administration, which has a low risk of injection site problems, low adverse events incidence, and is well tolerated [26]. Furthermore, incidences of injection-site hematomas have been reported to be very few. For instance, a study by Pini et al. rarely reported hematomas at injection sites in 187 patients receiving LMWH following DVT [27]. Moreover, a study by Blatter et al. [28] reported only a single injection site hematoma in 152 patients undergoing outpatient management of acute DVT.

While our research was developed to analyze recurrent VTEs among cancer victims taking either LMWHs or warfarin, prior research has compared data for thrombosis attributed to central venous catheters. This issue is crucial to cancer patients as a vast segment of cancer patients usually undergoes chemo or parenteral nourishment through long-term indwelling central venous catheters. A 1990 randomized study involving 82 patients with cancer on central venous catheters receiving either low-dose warfarin or placebo revealed that warfarin considerably decreased the occurrences of thrombosis than the placebo (p<0.001) but not the risk of hemorrhage, which was equivalent in both groups [29]. Another randomized study employing LMWH as the anticoagulant medication indicated that catheter-related thrombosis was considerably decreased in patients treated with LMWH than in those who failed to get prophylaxis (p = 0.002) [30]. Moreover, a 2003 open-label multicenter randomized study compared the antithrombotic effectiveness and safety of LMWH (nadroparin) with warfarin and showed that the frequency of thromboembolic episodes and fatality rates at six months did not vary between patients taking the two remedies [31]. Assuming the outcomes of this research are anything to go by, we can undoubtedly advise that LMWH can be taken as a substitute for cancer victims with contraindicated oral anticoagulant treatments.

While our study may suggest the benefit of LMWH over warfarin in reducing recurrent VTE, evidence from previous economic analyses has failed to demonstrate the cost-effectiveness of LMWH over warfarin. For instance, Marchetti et al. carried out a cost-effectiveness analysis for the secondary prevention of VTE and found that the long-term cost of LMWH was higher than that of warfarin ($904 vs. $667) [32]. Further analysis showed that 13 - 24% of the costs were attributed to the drug, 2 - 4% were due to treating bleedings and their sequels, and 74 - 83% were due to VTE treatment and its sequels. Similarly, a cost-effective analysis in Brazil showed that the monthly costs of warfarin were lower than that of LMWH for the management of cancer-related thrombosis ($1163.29 vs. $334.71, respectively) [33]. Moreover, Connell et al. concluded that even though LMWHs are associated with modest improvement in life expectancy, VKAs, including warfarin, are more cost-effective in managing cancer-related VTE than LMWHs [34]. This cost-effectiveness of warfarin may explain why it is still preferred for cancer patients with VTE, especially in low-income countries where the high cost of LMWH may result in an increased budget and affect their healthcare system. However, it is worth noting that the cost of LMWH may be lowered if patients are trained to self-inject in the course of the preliminary inpatient treatment of VTE because shorter hospital stays will be required afterward.

Evidence also suggests that warfarin may be associated with a reduction in new cancer incidences. Cancer incidence has been observed to rise within the first 12 months following VTE diagnosis and may grow at a minimum rate for at least the next 10 years [35,36]. A prospective multicenter randomized trial of 902 patients receiving warfarin for either six weeks or six months after the first episode of VTE reported a significantly lower incidence of newly diagnosed cancer in patients who received warfarin for six months than six weeks [n = 45 (10.3%) vs. n = 66 (15.8%), respectively; p = 0.02]. Further analysis in this study showed that disparity in the two arms was only noticeable in the second year of the follow-up, and the difference was only significant for urogenital cancer [n = 12 (2.8%) vs. n = 28 (6.7%, for patients receiving warfarin for six months and six weeks respectively; p = 0.01] [37]. Another large Norwegian cohort study reported that warfarin might have vast anticancer potential in patients older than 50 years [38]. According to this study, the cancer incidence among warfarin users was 9.4% compared to 10.6% in non-users. Further analysis showed that the difference was significant for prostate, lung, and female breast cancer. In addition, prior prospective research has evaluated the frequency of recently diagnosed clinically overt malignancy following the first case of idiopathic VTE. It showed no difference between patients who took oral anticoagulants after three months and those who took them after one year (6.2% vs. 8.7%, respectively) [39]. Evidence from additional research also shows that LMWHs may improve the outcomes of cancer due to their antithrombotic actions, suppression of coagulation proteases, and/or direct antitumor effects. Based on these investigations, it is evident that more randomized trials are necessary to properly demonstrate if anticoagulants have a clinically meaningful antineoplastic impact.

Limitations

Although most of the studies used in the current review were of high methodological quality, there were several limitations that should be taken into account when analyzing the results for clinical care. The most evident limitation of this review pertains to the eligibility criteria, which allowed the inclusion of articles published in English only. Although this criterion was essential in avoiding direct translations of scientific terms, results that would have otherwise added value to our analyses were not utilized. Our risk of bias assessment has also shown that attrition bias was present in some studies due to substantial withdrawals and termination rates. For instance, the study by Meyer et al. recruited few patients than initially planned [10]. According to this study, a study population of 120 patients in each cohort was necessary for the detection of the pre-planned decrease from 30% to 15% in the primary endpoints (recurrent VTE and or significant bleeding); however, the study was only able to recruit about 40% less than the expected sample size, thus limiting the ability to test its hypothesis. Similarly, Deitcher et al. had planned to recruit about 300 patients but only managed to recruit about 30% of this sample size [12]. This low sample size meant the study’s primary outcome (recurrent VTE) was undermined, and as such, our meta-analysis also might have been influenced. Furthermore, this study used two different treatment doses for LMWH. However, in our meta-analysis, we combined data for these doses into one arm, which might be a wrong decision if further randomized controlled trials on the outcomes of different LMWH doses are carried out. Finally, it is difficult to derive the effect of the study regimens on the outcomes of different cancer patients from our study because we could not carry out subgroup analyses based on various cancer types due to limited data.

## Conclusions

Our analysis suggests that LMWH is more effective than warfarin in reducing recurrent VTE among adult cancer patients. However, it has a similar effect on bleeding events and all-cause mortality as warfarin, meaning it is equally safe. Therefore, we recommend using LMWH as the first-line treatment regimen to prevent recurrent VTE in cancer patients. However, it is worth noting that the decision to start LMWH or warfarin in cancer patients with VTE should balance the benefits and shortcomings and patients’ values and preferences. Also, as shown in cost-effectiveness studies, LMWH is more expensive than warfarin and may be less preferred by patients due to its subcutaneous route of administration. Future research involving various subgroups of cancers to evaluate the effects of LMWH and warfarin for recurrent VTE in these subgroups would be highly beneficial. In addition, future studies should analyze the patient’s preferences and values regarding anticoagulation therapies to fully establish which treatment regimen should be recommended for cancer patients with VTE.
